# Epidemiology of Typhoid and Paratyphoid: Implications for Vaccine Policy

**DOI:** 10.1093/cid/ciy1124

**Published:** 2019-03-07

**Authors:** Senjuti Saha, Md Shfiqul Islam, Mohammad Saiful Islam Sajib, Shampa Saha, Mohammad Jamal Uddin, Yogesh Hooda, Md Hasan, Md Ruhul Amin, Mohammed Hanif, Mohammad Shahidullah, Maksuda Islam, Stephen P Luby, Jason R Andrews, Samir K Saha

**Affiliations:** 1 Child Health Research Foundation, Department of Microbiology, Dhaka Shishu Hospital, Dhaka, Bangladesh; 2 Department of Infectious Diseases, Stanford University School of Medicine, California; 3 Department of Biochemistry, University of Toronto, Ontario, Canada; 4 Bangladesh Institute of Child Health, Dhaka Shishu (Children) Hospital, Dhaka, Bangladesh; 5 Popular Diagnostic Center, Dhaka, Bangladesh; 6 Bangabandhu Sheikh Mujib Medical University, Dhaka, Bangladesh; 7 Shishu Shasthya Foundation Hospital, Dhaka, Bangladesh

**Keywords:** typhoid, paratyphoid, severity, vaccine policy, epidemiology

## Abstract

**Background:**

Typhoid and paratyphoid remain the most common bloodstream infections in many resource-poor settings. The World Health Organization recommends typhoid conjugate vaccines for country-specific introduction, but questions regarding typhoid and paratyphoid epidemiology persist, especially regarding their severity in young children.

**Methods:**

We conducted enteric fever surveillance in Bangladesh from 2004 through 2016 in the inpatient departments of 2 pediatric hospitals and the outpatient departments of 1 pediatric hospital and 1 private consultation clinic. Blood cultures were conducted at the discretion of the treating physicians; cases of culture-confirmed typhoid/paratyphoid were included. Hospitalizations and durations of hospitalizations were used as proxies for severity in children <12 years old.

**Results:**

We identified 7072 typhoid and 1810 paratyphoid culture-confirmed cases. There was no increasing trend in the proportion of paratyphoid over the 13 years. The median age in the typhoid cases was 60 months, and 15% of the cases occurred in children <24 months old. The median age of the paratyphoid cases was significantly higher, at 90 months (*P* < .001); 9.4% were in children <24 months old. The proportion of children (<12 years old) hospitalized with typhoid and paratyphoid (32% and 21%, respectively) decreased with age; there was no significant difference in durations of hospitalizations between age groups. However, children with typhoid were hospitalized for longer than those with paratyphoid.

**Conclusions:**

Typhoid and paratyphoid fever are common in Dhaka, including among children under 2 years old, who have equivalent disease severity as older children. Early immunization with typhoid conjugate vaccines could avert substantial morbidity, but broader efforts are required to reduce the paratyphoid burden.

Typhoid and paratyphoid, collectively known as enteric fever, are among the most common bacterial causes of morbidity worldwide, with the greatest burden in low- and middle-income countries [1]. *Salmonella enterica* subspecies *enterica* serovar Typhi (*Salmonella* Typhi), the causative agent of typhoid, is estimated to cause about 12 million illnesses and 128 000 deaths, and *Salmonella enterica* subspecies *enterica* serovar Paratyphi (*Salmonella* Paratyphi) A, B, and C, the causative agents of paratyphoid, are estimated to cause 4 million illnesses and 25 000 deaths annually [2, 3]. Recently, the World Health Organization (WHO) recommended typhoid conjugate vaccines (TCVs) in settings with high burdens and prequalified the first TCV [4]. There is no vaccine for paratyphoid. Countries are now facing important decisions regarding vaccine target groups and introduction strategies that require contemporary and locally relevant epidemiological data. Current estimates are primarily derived from sporadic, historical studies, and suffer from coarse geographical and temporal resolutions.

For effective implementation of the TCV, important questions regarding typhoid and paratyphoid epidemiology remain to be answered. For decades, typhoid was not considered severe enough in children under 2 years old to warrant treatment or hospitalization [5]. A few recent studies identified typhoid in young children, but severity in this age group remains largely unknown [6–8]. With the widespread use of antibiotics, case fatality rates and severe consequences of enteric fever, like intestinal perforation and peritonitis, have decreased significantly [9]. With low numbers of obvious, severe clinical outcomes, evaluating severity in endemic countries is difficult, yet necessary for informed policy decisions.

Typhoid and paratyphoid are considered a single disease, with the management of paratyphoid based on lessons learned from typhoid studies; yet, data on severities of illnesses from paratyphoid versus typhoid are conflicting [10, 11]. This is of specific interest in light of recent reports of increasing trends of paratyphoid from several countries, most of which have limited data sets [12, 13]. Most epidemiological data are obtained from hospital inpatient departments (IPD), but in endemic countries, a large number of patients seek care and are treated at outpatient departments (OPD) [14–16].

We initiated enteric fever surveillance in 2004 in Bangladesh, where no typhoid vaccines are used. The surveillance was conducted in the IPDs of the 2 largest pediatric hospitals and the OPDs of the largest pediatric hospital and a private consultation clinic. We describe the (1) trend in typhoid and paratyphoid cases, (2) age distribution of cases, and (3) severity by age of laboratory-confirmed cases of typhoid and paratyphoid, using hospitalizations and durations of hospital stays as proxies for severity.

## METHODS

### Study Sites, Population, and Case Definitions

This study included 3 sites in Dhaka, Bangladesh: (1) Dhaka Shishu Hospital (DSH), (2) Shishu Shasthya Foundation Hospital (SSFH), and (3) an outpatient-based diagnostic center, the Popular Diagnostic Center (PDC). With 640 beds, DSH is the largest pediatric hospital in Bangladesh, and provides primary to tertiary care to patients up to 18 years of age; 47% of patients are treated free of cost. SSFH is the second-largest pediatric hospital, with 200 beds for children up to 14 years of age; about 5% of the beds are dedicated to those unable to pay. PDC is among the largest private consultation centers, and serves patients of all ages.

For IPD-based surveillance to capture hospitalized enteric fever cases, we included cases from the IPDs of both DSH and SSFH. For OPD-based surveillance, we included cases from the OPDs and emergency rooms of both DSH and PDC. Blood cultures were conducted at the discretion of the treating physicians. We enrolled patients with positive blood cultures for *Salmonella* Typhi or Paratyphi.

### Etiology Detection

Blood cultures were performed using standard methods [17]. We aseptically obtained 2–3 milliliters of blood, which was inoculated into trypticase soy broth supplemented with sodium polyanethole sulphonate (0.25%) and isovitalex (1%). Incubated blood culture bottles were sub-cultured on the second, third, and fifth days of incubation. Identification of *Salmonella* Typhi/Paratyphi isolates was confirmed using standard biochemical tests and agglutination with *Salmonella* species and serovar-specific antisera (Ramel, Thermo Fisher Scientific). Laboratory methods for blood culture and organism identification were consistent over the reporting period.

### Data Collection and Analysis

For enrolled cases, we collected the patient’s age, the presence of hospitalization, the duration of hospital stay, and hospital outcomes from hospital and clinic records. We analyzed the proportion of typhoid and paratyphoid cases, the age distribution, and the number of patients hospitalized. Many caregivers in Bangladesh do not know the exact age of the child, and round up to the closest year. To assess the age distributions of cases under 5 years, age smoothing was conducted. For children over 12 months old, the exact age was not always known, but we used the date of birth of the child if it was available. The remaining cases with estimated ages were allocated evenly across the months of that year, or “smoothed.” We used hospitalizations and durations of hospitalizations as proxies of disease severity. The age distribution of the care-seeking populations varied between our study sites, as DSH and SSF are pediatric hospitals and PDC provides care to all age groups. In the hospitals, >95% of cases are in patients <144 months old (12 years old); therefore, we limited the analyses on the severity of typhoid and paratyphoid to only cases of children <12 years old. We used the Chi-square test to assess whether the proportion of typhoid/paratyphoid cases was progressively increasing or decreasing and the risks of hospitalization with age. We used the Wilcoxon rank-sum test (or Kruskal–Wallis test, depending on the group numbers) for assessing the differences in age distributions of typhoid/paratyphoid cases treated in the OPD and IPD and for differences in the durations of hospitalization between typhoid/paratyphoid cases and in different age groups of typhoid/paratyphoid. We used the z-test for evaluating differences in the proportion of hospitalized typhoid and paratyphoid cases in children under 12 years old.

### Ethical Clearance

The protocols were approved by the ethics review committees of the Bangladesh Institute of Child Health, DSH. Blood samples were collected and received at the laboratory as part of routine clinical care, and informed consent was obtained from parents or caregivers for other aspects of the study, including data collection and the use of specimens for additional laboratory analyses.

## RESULTS

### Proportion of Typhoid and Paratyphoid Fever Cases

**Figure 1.**
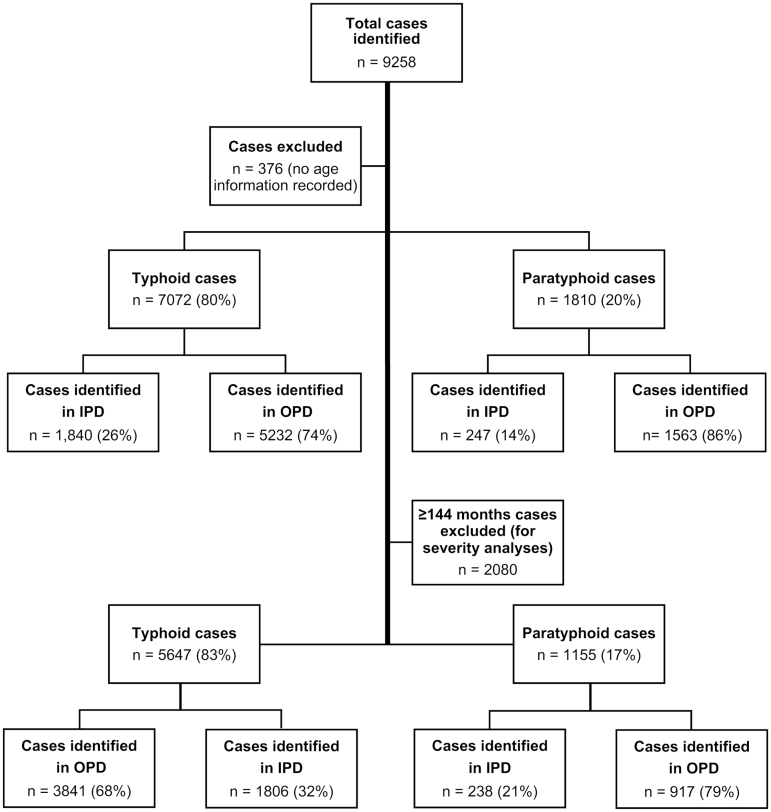
Typhoid and paratyphoid cases enrolled in this study. Abbreviations: IPD, inpatient departments; OPD, outpatient departments.

**Figure 2.**
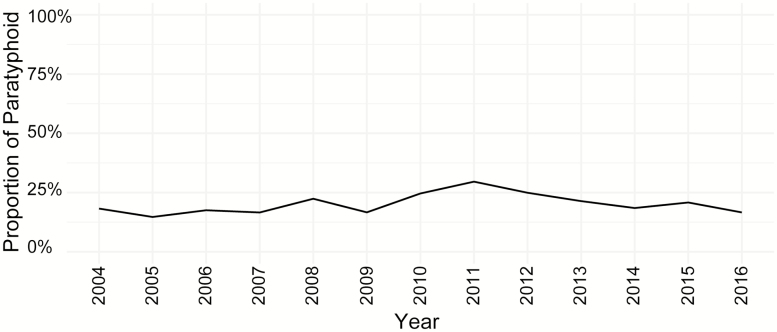
Proportion of paratyphoid cases within all enteric fever cases in Bangladesh, 2004–2016.

From 2004 through 2016, we identified 7072 cases that were blood culture–positive for *Salmonella* Typhi and 1810 cases that were positive for *Salmonella* Paratyphi A, with relevant clinical data for downstream analyses (Figure 1). In total, 60% (5296/8882) of the cases were enrolled in an outpatient-based diagnostic center, PDC, which serves patients of all age groups. During this period, Paratyphi A accounted for 20% of the typhoidal *Salmonella* isolates. While this fluctuated moderately from year to year (peak of 30% in 2011, trough of 15% in 2005), there was no increasing or decreasing trend over the 13-year study period (Figure 2).

### Age Distribution and Estimated Vaccine Coverage

**Figure 3.**
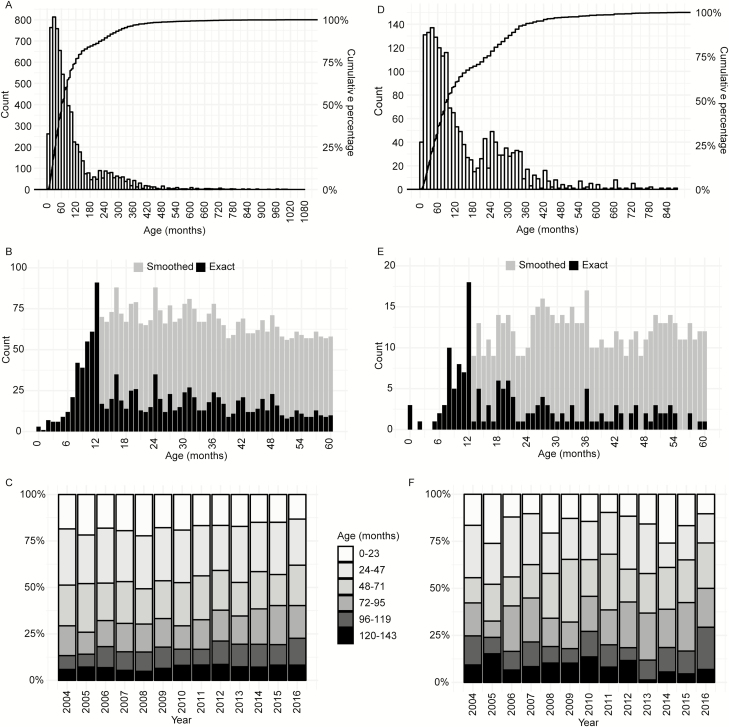
Age distribution of enteric fever cases in Bangladesh. *A*, Age distribution of all typhoid cases. *B*, Age distribution of typhoid cases in children under 5 years old. For children above 12 months old, the exact age, shown in black, was known only in some cases, where the exact date of birth of the child was available. The remaining cases, where the exact birth date was unavailable, and approximate age was recorded, were allocated evenly across months of that year, or “smoothed,” shown in gray. *C*, Proportion of typhoid cases in different age groups each year; only cases of children <12 years old were included here. *D*, Age distribution of all paratyphoid cases. *E*, Age distribution of paratyphoid cases in children under 5 years old. *F*, Proportion of paratyphoid cases in different age groups each year; only cases in children <12 years old were included here.

There were 3 children <1 month old who had blood culture–confirmed typhoid. The oldest case was in a 90-year-old patient; 15% (1025/7072) of cases were in children <24 months old and 46% (3251/7072) in children <60 months old (Figure 3A). Only 1.5% (107/7072) cases occurred in children <9 months of age (Figure 3B). While the median age of typhoid cases among children was 60 months (interquartile range [IQR] 33–120) and a peak was seen between 36 and 60 months, a second, smaller peak was apparent at 216 to 264 months (18 to 22 years; Figure 3A).

The youngest paratyphoid patients were <1 month old (n = 3) and the oldest was 73 years old; 9.4% (171/1810) of cases were in children <24 months old and 31.5% (570/1810) were in children <60 months old (Figure 3D and 3E). The median age of all cases was 90 months (IQR 48–228 m), compared with 60 months in typhoid cases (*P* < .001). Similar to typhoid, we also observed a bimodal age distribution among the paratyphoid cases. The peaks here were broader, where the first peak occurred at 12 to 84 months and the second peak at 204 to 336 months (17 to 28 years).

The overall age distributions of both typhoid and paratyphoid cases in children <12 years old remained fairly consistent through the 13 years of our surveillance (Figure 3C and 3F).

### Severity of Typhoid and Paratyphoid by Age

Hospital outcomes were available for 1188 typhoid cases: 2 (0.2%) case patients died, 36 (3%) left against medical advice, 4 (0.3%) were referred to other hospitals, and 1146 (96.5%) were discharged. Of the 164 paratyphoid cases for which hospital outcomes were available, 7 (4.2%) left against medical advice and the remaining 157 (95.8%) were discharged. No deaths among paratyphoid-infected patients were confirmed. As severe complications or deaths due to enteric fever were uncommon, it was not possible to perform robust statistical analyses to compare severities of the diseases in different age groups. Therefore, we used hospitalizations and durations of hospitalizations as proxies of severity in children <12 years old. This data set consisted of 5647 typhoid cases (median age 48 months, IQR 28–78) and 1155 paratyphoid cases (median age 60 months, IQR 33–84 months; *P* < .001).

### Hospitalization as a Proxy for Severity

**Figure 4.**
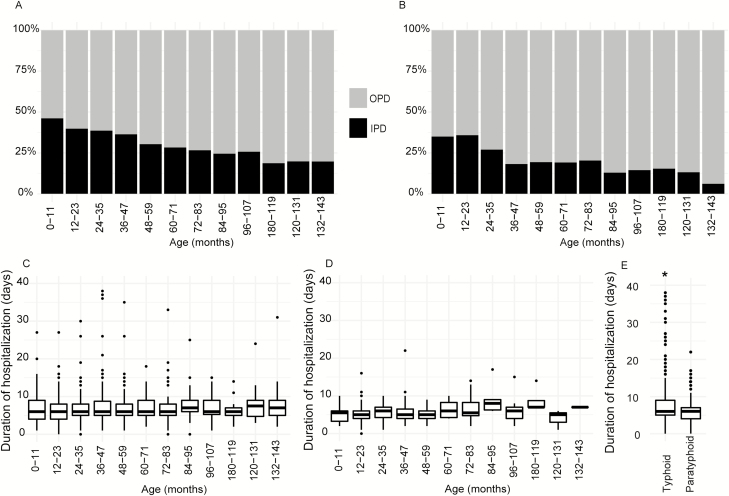
Severity of enteric fever with age. *A*, Proportion of typhoid patients hospitalized in the IPD (black), compared to proportion of typhoid patients treated in the OPD (gray), in each age group. *B*, Proportion of paratyphoid patients hospitalized in the IPD (black), compared to proportion of paratyphoid patients treated in the OPD (gray), in each age group. *C*, Distribution of durations of hospital stays of typhoid cases in each age group. *D*, Distribution of durations of hospital stays of paratyphoid cases in each age group. *E*, Distribution of durations of hospital stays of both typhoid and paratyphoid cases. Abbreviations: IPD, inpatient departments; OPD, outpatient departments. *Designates a significant difference of *P* < .001 (Wilcoxon Rank-Sum test or Kruskal-Wallis tests were performed, depending on the group numbers).

Among the 5647 typhoid patients under 12 years old, 1806 (32%) were hospitalized and the remaining 3841 (68%) were treated in an OPD. By age, 41.5% (425/1025) of children <2 years old were hospitalized, compared to 35% (789/2226) of children 2–5 years old and 24.7% (592/2396) of those 5–12 years old. Younger children were more likely to be hospitalized; the proportion of children hospitalized with typhoid fever decreased with age (*P* < .001; Figure 4A). Overall, the median age of the hospitalized children was 42 months (IQR 24–69), compared to 54 months (IQR 30–84) for children treated in an OPD (*P* < .001).

For 1155 paratyphoid cases, the proportion of children hospitalized decreased with increasing age (*P* < .001; Figure 4B); 35.7% (61/171) of children <2 years old were hospitalized, compared to 21.6% (86/399) of children 2–5 years old and 15.6% (91/585) of children 5–12 years old. The median age of hospitalized paratyphoid cases was 46 months (IQR 22–72) and of OPD cases was 60 months (IQR 36–87; *P* < .001). Compared to typhoid, there was a lower overall proportion of paratyphoid cases hospitalized (21% [238/1155] compared to 32%; *P* < .001).

### Hospital Duration as a Proxy for Severity

Hospital duration data were available for 1217 of 1806 hospitalized typhoid cases, with a median of 6 days (IQR 5–9). There was no significant difference in the durations of hospitalization with age (Figure 4C). For paratyphoid cases, the durations of hospital stays were available for 161 of 238 hospitalized cases, and the median duration was also 6 days (IQR 4–7), with no significant difference in the durations of hospitalization with age (Figure 4D). Although the median durations of hospitalization of typhoid and paratyphoid cases did not differ, a Wilcoxon rank-sum test showed significant difference in the distribution of durations of hospitalization (*P* < .001); typhoid cases were more likely to remain hospitalized longer than paratyphoid cases (Figure 4E).

## DISCUSSION

Most low- and middle-income countries have introduced, or are in the process of introducing, the pneumococcal conjugate vaccine and *Haemophilus influenzae* type b vaccine, based on relevant burden and vaccine efficacy data, which has significantly decreased the burden of pneumococcal and *Haemophilus influenzae* type b diseases. However, it remains difficult to make evidence-based decisions on the control of enteric fever, as only a few, small, incomprehensive studies have addressed disease epidemiology. With the recent WHO prequalification of TCV, a detailed understanding of typhoid in infants and young children is important for developing the most effective vaccination strategy. We conducted enteric fever surveillance from 2004 through 2016 to generate data on typhoid and paratyphoid cases in Bangladesh, where *Salmonella* Typhi and Paratyphi A are the most common causes of bloodstream infections in children over 2 months of age [17, 18].

In recent years, there have been several reports of increasing proportions of paratyphoid, as compared to typhoid [12, 13]. Our surveillance revealed no trend of change, corroborating results from a meta-analysis of data from India [19]. We did observe moderate year-to-year fluctuations in the proportions of the 2 diseases, which could be due to a number of factors, including changing patterns in the antimicrobial susceptibility of *Salmonella* Typhi and Paratyphi A or other environmental factors. For example, in Bangladesh, the multidrug resistance (resistance to ampicillin, cotrimoxazole, and chloramphenicol) of *Salmonella* Typhi has significantly decreased in recent years; there is no multidrug-resistant Paratyphi A strain [14]. Almost all *Salmonella* Typhi and Paratyphi A strains are non-susceptible to fluoroquinolones [17]. As antibiotic consumption prior to seeking care is very common, the presentation of cases at a facility depends on the available antibiotics in the market, dynamics of antimicrobial susceptibility patterns of the organisms, and rates of treatment failure in the community [15].

In concordance with the few studies that looked at the prevalence of typhoid in children <5 years old, here 15% of cases were in children <2 years old and 46% were in patients <5 years old. In a study conducted in 2001, through a year of surveillance with 391 typhoid cases, our group had demonstrated that 27% of typhoid fever cases were in children <2 years old and 54% were in patients <5 years old [7]. The proportions depicted in the current study are lower than our previous studies, probably because we included both pediatric hospitals and private consultation centers that serve all age groups. The findings are similar to the disease burden data among a preschool children group in India (44% were <60 months old; n = 63 cases) and in a slum population of Bangladesh (60% were <60 months old; n = 49 cases) [6, 20].

The WHO has recommended TCV in children as young as 6 months and adults up to 45 years living in typhoid endemic areas, with an emphasis on the routine, programmatic administration of TCV at the same time as other vaccine visits at 9 months of age or by 2 years of age [4]. Our data on the age distribution of typhoid cases illustrate that if TCV is administered at (1) 6 months, 0.5% of the cases will be missed, (2) if at 9 months, 1.5% will be missed, or (3) if at 24 months, 15% will be missed. These findings emphasize the need for starting vaccination as early as 6 or 9 months.

We also noticed a rise in the number of cases in adults aged 18 to 22 years, which is probably due to this population leaving home to start undergraduate studies or jobs and having increased exposure to outside food. This bimodal peak suggests that a catch-up vaccination program could reduce the population burden and may decrease overall transmission.

In our study, children with paratyphoid were generally older than children with typhoid (median age 90 months vs 60 months, respectively), but 9.4% and 31.5% of paratyphoid cases occurred in children under 2 and 5 years old, respectively, with a similar peak in young adults. A study conducted in a general hospital in Nepal that serves patients of all ages also depicted a higher median age of paratyphoid patients, compared to typhoid patients [21].

In Bangladesh and the surrounding region, most health-care costs are paid out of pocket and, therefore, the most impoverished seek care only when absolutely necessary; most enteric fever cases are empirically treated at the OPDs of hospitals or community clinics [22]. Patients are admitted in an IPD only when they are very sick and/or are not responding to treatment. Hospitalized patients are eager to leave when they feel slightly better and/or are no longer able to afford the costs [22]. Furthermore, amidst fierce competition for beds, hospital physicians advise OPD-based treatment if the case is not considered severe enough to require hospitalization [23]. Based on these accounts and the rarity of known complications of enteric fever, we selected hospitalizations and durations of hospital stays as proxies of severity.

The proportion of children hospitalized with typhoid decreased with age, and younger children were significantly more likely to be hospitalized. While it is possible that younger children are more likely to be hospitalized than older children with the same degree of severity of the disease, the data delineate that typhoid is definitely not less severe or benign in young children. There was no difference in the durations of hospital stays between age groups, further illustrating equal severities of disease cases across age groups. Paratyphoid cases are less likely to be hospitalized than typhoid cases (21% vs 32%, respectively), and those paratyphoid patients who are hospitalized are likely to stay for a shorter duration. Although paratyphoid appears less severe than typhoid and there was no increase over the surveillance period, it is still an important disease that frequently affects young children, leads to the hospitalization of one-fourth of the affected children, and, hence, merits immediate action on prevention and control.

An important limitation of the study is that it was not designed to actively identify suspected enteric fever cases. The use of blood cultures was dependent on the treating physicians: it likely differed between facilities and may have biased age patterns and the proportion of typhoid and paratyphoid cases. Our study included hospitalized typhoid/paratyphoid cases only from pediatric hospitals and severity analyses were conducted only in children <12 years old. Further work is required to understand and compare the severities of the diseases in older age groups.

Important strengths of our study include (1) the large number of culture-proved cases, which allowed for robust analyses of age distributions, (2) analyses of both proportions of hospitalizations and durations of hospital stays for the assessment of severity, and (3) a long surveillance period, with data collection for many years, which confirmed consistency instead of providing snapshots of the disease. This knowledge is crucial, both to control the diseases with appropriate utilization of interventions and as baseline data for future vaccine impact studies. Monitoring the age distribution of typhoid cases and the burden of paratyphoid will be important following the introduction of TCVs.

## CONCLUSIONS

With 8882 cases of enteric fever identified from 2004 through 2016, our findings demonstrate that typhoid and paratyphoid are common in young children. Although the case fatality rate is low, the emergence of widespread antimicrobial resistance is threatening effective treatment and, if the right intervention strategies are not instituted soon, increases in mortality rates in the near future may occur. We found that 15% of cases occur in children under 2 years old, with equivalent disease severity as seen in older children. Early immunization with TCVs could avert substantial morbidity. Efforts to reduce exposure to contaminated water and food, as well as developing vaccines against paratyphoid, are important steps for the prevention and control of enteric fever.

## Notes

***Acknowledgments.*** The authors thank Mr Hafizur Rahman for his technical assistance in the laboratory.

***Financial support.*** The work presented here was supported by the Gavi, the Vaccine Alliance, through the World health Organization-supported Invasive Bacterial Vaccine Preventable Diseases study (grant numbers 201588766, 201233523, 201022732, 200749550) and through the Pneumococcal Vaccines Accelerated Development and Introduction Plan (PneumoADIP) and by the Child Health Research Foundation.

***Supplement sponsorship.*** This supplement is sponsored by the Center for Vaccine Development and Global Health (CVD) at the University of Maryland School of Medicine.

***Potential conflicts of interest.*** All authors: No reported conflicts. All authors have submitted the ICMJE Form for Disclosure of Potential Conflicts of Interest. Conflicts that the editors consider relevant to the content of the manuscript have been disclosed.
